# Forage quality shapes physiological and gut microbial responses in moose (*Alces alces*) of Isle Royale National Park

**DOI:** 10.1038/s41598-026-35555-w

**Published:** 2026-01-27

**Authors:** Sebastian Menke, Gloria Fackelmann, Leah M. Vucetich, John A. Vucetich, Jennifer S. Forbey, Simone Sommer

**Affiliations:** 1 Institute of Evolutionary Ecology and Conservation Genomics, University of Ulm, Albert-Einstein Allee 11, 89069 Ulm, Germany; 2 Department of Cellular, Computational and Integrative Biology, University of Trento, 38122 Trento, Italy; 3 School of Forest Resources and Environmental Science, Michigan Technological University, 1400 Townsend Drive, Houghton, MI 49931 USA; 4 Department of Biological Sciences, Boise State University, 1910 W University Dr, Boise, ID 83725 USA

**Keywords:** Moose, Balsam fir, Herbivory, Plant secondary compounds, Detoxification, Microbiome, Metagenome, Terpenes, Ecology, Ecology, Microbiology

## Abstract

Plant secondary compounds (PSCs) impose physiological and nutritional constraints on herbivores, yet many species continue to rely on PSC-rich forage during critical periods of the year. Moose (*Alces alces*) on Isle Royale National Park depend heavily on balsam fir (*Abies balsamea*) during winter, exposing them to a chemically defended and nutritionally limited diet. To investigate how this foraging strategy shapes both physiological responses and gut microbial communities, we integrated fecal diet composition, urinary markers of detoxification and nutritional status, 16S rRNA gene sequencing, and shotgun metagenomic profiling from free ranging moose of two separated populations from the western and eastern region of the island. Balsam fir consumption varied strongly by region and was positively associated with glucuronic acid to creatinine (GA:C) and urea nitrogen to creatinine (UN:C) ratios, indicating increased detoxification activity and reduced nutritional condition. Microbial alpha diversity declined with higher fir intake in nutritionally limited individuals, while beta diversity differed by region, balsam fir consumption, and UN:C. Several bacterial genera responded to PSC exposure, including increases in the butyrate-producing genus *Roseburia* and shifts in network prominence of *Phascolarctobacterium*. Metagenomic data revealed pathways involved in the degradation of aromatic and terpenoid PSCs, although pathway abundances did not differ significantly with balsam fir consumption after multiple testing correction. These results show that winter foraging on balsam fir produces coordinated dietary, physiological, and microbial patterns, with both host and gut microbial detoxification capacities interacting to accommodate the chemical and nutritional challenges of a PSC-rich winter diet.

**Supplementary Information:**

The online version contains supplementary material available at 10.1038/s41598-026-35555-w.

## Introduction

 Many plant species have evolved chemical defense mechanisms, known as plant secondary compounds (PSCs), to deter herbivory. These compounds are synthesized by the plant secondary metabolism, which, unlike the plant primary metabolism, is not directly involved in plant development and growth^1^. Secondary metabolism provides plants with a diverse array of PSCs to defend against herbivory^2,3,4^. The three main groups are phenolics, terpenoids and alkaloids. The wide range of chemical structures across the groups is associated with a correspondingly broad spectrum of adverse effects in herbivores following ingestion, including disruption of cell membranes, tissue damage, generation of free radicals, neurotoxicity, hepatotoxicity and carcinogenic effects, with death being the most severe outcome ^5^.

In response, herbivores have developed a variety of behavioral and physiological mechanisms to counteract PSCs. Behaviorally, herbivores can limit toxic concentration of PSCs by regulating intake through altered meal size and frequency^6,7^ and diet mixing^8^. After ingestion, tannin-binding salivary proteins discovered in several mammal species^9–11^, including moose (*Alces alces*)^12^, can prevent ingested tannins from reducing protein digestion. Several animals, including parrots, elephants and primates, may use geophagy to bind ingested PSCs to prevent their bioactivity or absorption^13-15^. In addition, efflux transporters in the gut can limit absorption^16^ but are energetically expensive^6,17^. Detoxifying enzymes in the liver convert ingested PSCs into more water-soluble metabolites, facilitating their excretion from the body via the urine^18,19^. Furthermore, the co-evolution of plants and herbivores has led to genetic diversification of host genes^20,21^ involved in metabolic detoxification, which helps herbivores mitigate the negative effects of PSC intake^21,22^. All these mechanisms prevent herbivores from overloading detoxification pathways^23^ which serve as the key physiological mechanism by which herbivores reduce exposure to ingested and absorbed PSCs.

Other studies have highlighted the significance of the gut microbiome for herbivores consuming plants rich in PSCs, as they provide additional metabolic pathways for PSC detoxification^24^. For instance, the gut microbiome of the cabbage root fly possesses plasmids encoding a gene responsible for catalyzing the conversion of a plant toxin^25^. Noteworthy research on dietary specialists has underscored the pivotal role of gut bacteria in overcoming high levels of PSCs in their primary diet^26–28^. Consequently, gut microbes possess the capacity to expand the dietary niche of mammalian herbivores^29^, enabling them to consume food sources that would otherwise be toxic^30^. Gut microbes, on the other hand, benefit from the host by gaining access to substrates required for their metabolism.

Building on this understanding of microbial contributions to PSC metabolism, we examined these processes in a natural system where herbivores experience pronounced spatial variation in availability of a major food source rich in PSC. Isle Royale National Park (IRNP), North America, provides such a setting, with moose (*Alces alces*) populations inhabiting regions that differ markedly in the abundance of balsam fir (*Abies balsamea*), a conifer tree species rich in PSCs. While the occurrence of balsam fir has declined markedly in the western region of the island, it has remained relatively stable in the eastern part^31–33^. Correspondingly, previous studies have reported an east-west division in the proportion of balsam fir in moose diet^32^. Moose rely heavily on woody browse during the winter, including balsam fir despite its high content of PSCs^32,33^. The juxtaposition of a major herbivore species with a dominant winter food source that varies spatially in abundance creates a unique opportunity for examining how differences in exposure to PSCs shape the physiology and gut microbiome of a large herbivore. Thus, to improve our understanding of how dietary PSCs shape both host physiological responses and gut microbial dynamics in moose, we used an integrative approach that combined ecological, nutritional, physiological, and gut microbiome data within this natural experimental setting.

Specifically, we:

1. Collected demographic data of moose including age and sampling location.
2. Analyzed the diet composition of individual moose to understand the relative proportions of plant species consumed using microhistology of food content in moose fecal samples.
3. Measured the glucuronic acid to creatinine ratio (GA:C) as a measure of detoxification status and the urinary urea nitrogen to creatinine ratio (UN:C) in snow urine samples as an indicator of nutritional status.
4. Analyzed moose fecal bacterial 16S rRNA gene and shotgun metagenomics sequences to investigate how gut microbial community composition and metagenomic functional profiles were influenced by both intrinsic (physiological) and extrinsic (environmental) factors.

Given the strong west-east differences in balsam fir availability on IRNP, we hypothesized that moose in the eastern region would experience greater exposure to PSC rich forage and therefore exhibit higher detoxification investment, reduced nutritional status, and corresponding shifts in gut microbial community composition and metabolic pathways associated with PSC degradation.

## Materials and methods

### Sampling of moose feces and urine

**Fig. 1:**
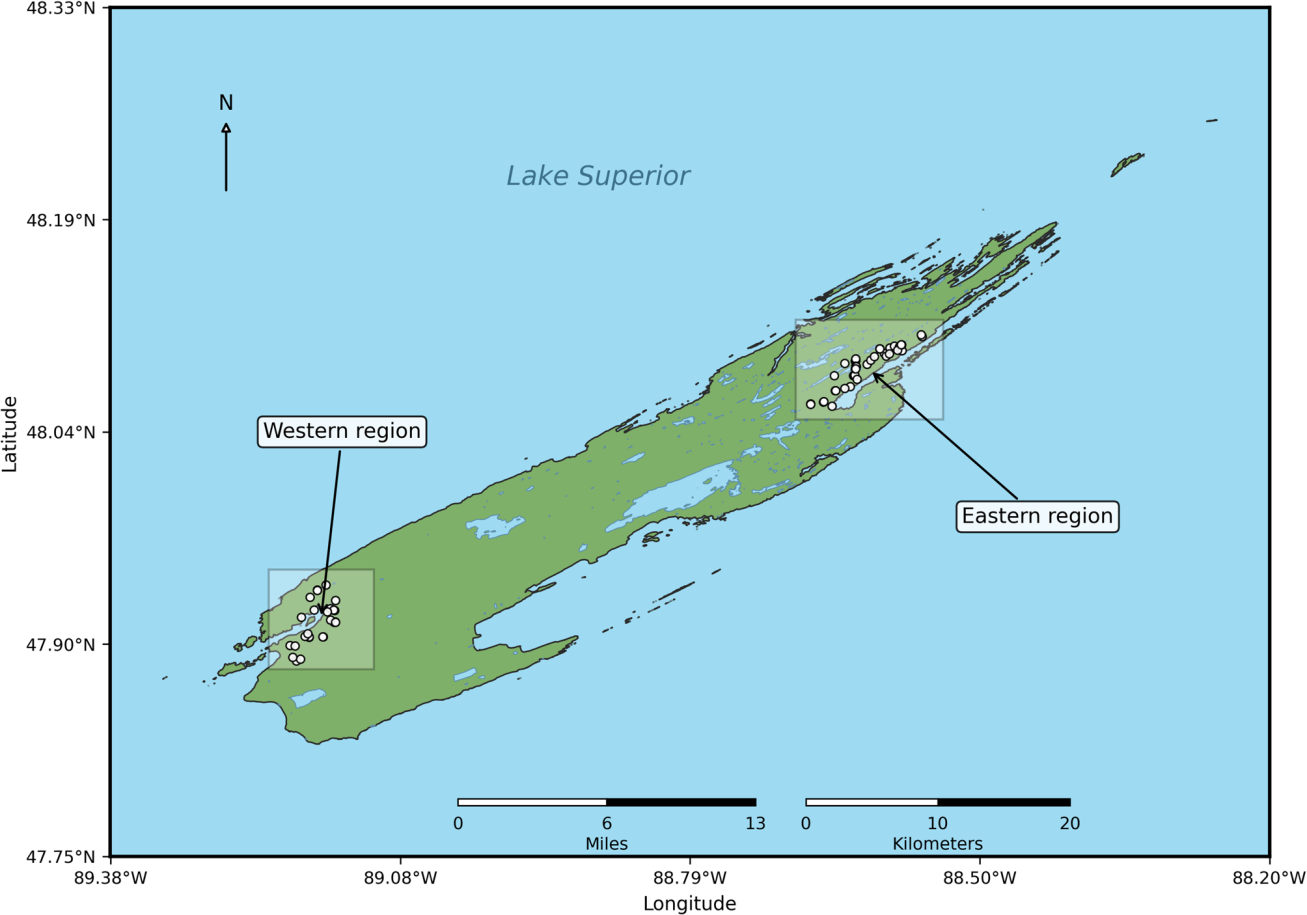
**Map of Isle Royale National Park.** Shaded areas represent the western and eastern moose sampling regions and circles mark the individual moose sampling sites.

71 moose fecal pellets were collected in the eastern (*n* = 45) and western (*n* = 26) region of IRNP during a narrow four-week sampling period during winter of 2017 (Fig. 1). Sampling locations within each region were sufficiently spaced to make sure that samples came from different moose individuals. The park encompasses 544 km² and has supported a moose population since the early 20th century^32^. During the year of sample collection (2017), the population was estimated at approximately 1,600 individuals^32^. The eastern and western sampling regions were seperated by 45 km, a distance sufficient to ensure that no sampled moose was represented in both regions. To minimize environmental bacterial contamination, fecal samples were collected from the core of the pellets under sterile conditions. Samples were then stored in RNAlater, incubated for 24 h and frozen at -20 °C until DNA isolation. In addition to fecal samples, urine was collected following voluntary release into the snow; only snow urine samples paired with fecal samples from the same location and moose individual were included in the analyses.

### Diet composition

To determine the diet of each individual moose, microhistology of food content was analyzed from the fecal samples. Briefly, moose fecal samples were dried, ground, drained, bleached and fixed on microscope slides. Using a light microscope and a predefined number of grids, plant material was compared to a curated reference collection, and the proportion of each plant species was determined. A more detailed description can be found in Parikh^34^.

### Physiological status of moose

To determine the physiological status of moose, measurements of urea nitrogen (UN), creatinine (C) and glucuronic acid (GA) were taken from urine samples as detailed in Parikh et al. (2017). UN:C ratios represent our measure of nutritional status of moose. UN:C ratios greater than 3.5 are associated with increased catabolism of endogenous protein, indicating starvation and severely reduced nutritional status in ungulates^35^. Additionally, prolonged low UN:C ratios in wild populations may reflect increased renal reabsorption of urea nitrogen, signaling chronic nutrient limitation that could precede dangerously high UN:C ratios (> 3.5)^36^. GA:C ratios represent our measure of detoxification status. GA is a urinary metabolite that is related to the amount of toxin that is consumed, absorbed, and metabolized by the glucuronidation pathway^37^. Higher GA:C therefore represents higher intake of PSCs by moose and is associated with reduced nutritional status (i.e., higher UN:C) in moose at IRNP^32,38^. To measure UN:C and GA:C ratios, snow urine samples were melted and analyzed using spectrophotometry (UN and C) and colorimetry (GA). Each sample was measured twice, and the results were averaged.

### Microbiome sequencing

Prior to DNA extraction, samples were pre-treated to remove excess RNAlater and to enrich bacterial DNA relative to non-bacterial DNA. This was achieved by first transferring 200 mg of fecal matter into 1.5 mL Eppendorf tubes using a spatula. 400 µL ice-cold PBS (phosphate-buffered saline)^39^ was then added to each individual tube and the mixture vortexed for 10–15 s, after which the samples were centrifuged (Centrifuge 5424, Eppendorf) at 700 rpm for two min. The supernatant was then transferred to a new 1:5 mL Eppendorf tube (avoiding the transfer of pelleted plant material), and centrifuged again, this time at 14,000 rpm for 10 min. After discarding the supernatant, the remaining pellet was used as the starting material for DNA isolation with the commercially available NucleoSpin^®^Food kit from Macherey-Nagel. An additional bead-beating step was incorporated between the step of chemical lysis of sample cells with the provided CF Buffer and the DNA binding adjustment step. This was done to mechanically lyse bacterial cells present in the samples by transferring the solution from the lysis step to tubes with ceramic beads and subsequently beating the solution twice at 20 s per round. After centrifugation at 11,000 rpm for two min., the clear supernatant was transferred into 2 mL Eppendorf tubes before proceeding with the DNA binding adjustment step of the protocol. For normalization of sample DNA concentrations, each sample was measured on the TECAN infinite F200PRO fluorescence plate reader (serial number: 1701007484).

#### 16S rRNA gene high-throughput sequencing

The Illumina MiSeq DNA Amplicon sequencing library was prepared using polymerase chain reaction (PCR) using Fluidigm chemistry (Fluidigm Corporation, South San Francisco, CA, USA) and PCR protocol. To amplify the V4 target region of the 16S rRNA, the primers 515F ( 5′-GTGCCAGCMGCCGCGGTAA-3′ ) and 806R ( 5′-GGACTACHVGGGTWTCTAAT-3′) were used^40^. These primers were adapted to the Fluidigm system and equipped with a tail that is complementary to parts of the Illumina sequencing adapters resulting in 515 F-CS1 and 806R-CS2. All PCRs were performed with a total volume of 20 µl containing 3 µl H_2_O, 10 µl AmpliTaq Gold^®^ 360 Master Mix, 2 µl TS primer mix (96 µl H_2_O, 2 µl primer 515 F-CS1, and 2 µl primer 806R-CS2), 3 µl of barcode-containing sequencing adapter, and 2 µl DNA sample. The PCR was run on a SimpliAmpTM Thermo Cycler (Fisher Scientific) with a complex cycling protocol by which the DNA is first amplified and subsequently equipped with Illumina-compatible sequencing adapters.

Before normalization, samples were cleaned using the ‘NucleoMag^®^ NGS Clean-up and Size Select Kit’ (Machery & Nagel, Düren, Germany) with the ‘Gene Theatre Robot’ (Analytic Jena) using the program system program ‘Application Studio’ and the clean-up program ‘Variable Volume 2018’. The DNA concentrations of cleaned samples were measured on the ‘Qiagen Qiaxcel Advanced’ with an alignment marker ranging from 15 to 1000 bp and a size marker ranging from 50 to 800 bp using 5 ng/µl each. Finally, samples were pooled at an equimolar amount of DNA (3 ng), again using the ‘Gene Theatre Robot’ and transferred to a 1.5 µl Eppendorf tube. DNA concentration of the pool was measured on the ‘TECAN infinite F200PRO’ fluorescence reader (serial number: 1701007484) to prepare the final concentration of 6 nM.

For sequencing of the 16S rRNA gene sequencing library on Illumina, the ‘protocol A’ of the ‘Illumina^®^MiSeq System Denature and Dilute Libraries Guide’ (15039740) was applied. As an additional step and after incubation of the sequencing library and PhiX control (Illumina^®^, San Diego, CA, USA) at room temperature, both tubes were incubated at 95 °C in a thermoshaker for 2 min. Then, the library and the PhiX control were combined resulting in a proportion of 5% PhiX. Finally, the sequencing library was loaded in the reagent cartridge (MiSeq^®^ Reagent Kit v2 (500 cycle) MS-102-2003) and a paired-end read was performed. We sequenced 18 blanks to control for contamination following the same sample preparation.

FASTQ files from the Illumina MiSeq run were processed using ‘Quantitative Insights Into Microbial Ecology 2’ (QIIME 2)^41^. First, the demultiplexed FASTQ files and metadata were imported into QIIME 2 and converted into an artifact file. Next, using dada2^42^, sequencing reads were quality filtered, primers were trimmed (p-trim-left-f 23, p-trim-left-r 20), chimera were removed, and final sequencing reads were collapsed into amplicon sequence variants (ASVs).

Blanks showed very little contamination (min reads: 0, max reads: 116, mean: 24.11, median: 17.50, SD: 29.31). We removed those ASV from all samples but protected the 20 most abundant ASVs. In addition, we removed moose samples of insufficient sequencing depth and dropped archaeal, chloroplast and mitochondrial sequences. Subsequently, sequences were aligned to generate a midpoint-rooted phylogenetic tree for microbial diversity analyses and taxonomy was assigned to ASVs using a pre-trained native Bayes classifier trained on the Greengenes 13_8 99% OTUs^43,44^. For downstream analyses, we imported the taxonomy-containing biom table, a phylogenetic tree, and the reference sequences into R^45^. Finally, we added a metadata file and combined all into a *phyloseq* object^46^. The final ASV table consisted of 66 moose samples with total of 3,852,703 reads (58,374 ± 23,644) and 3,329 bacterial taxa.

#### Shotgun metagenomic sequencing

To obtain gut microbial functional information, we sequenced isolated DNA from fecal samples using a metagenomic shotgun approach using the ‘Nextera^®^XT DNA Library Preparation Kit’ from Illumina^®^. In brief, an enzymatic reaction cuts DNA into fragments of approximately 300 bp, whilst simultaneously tagging these fragments with oligonucleotides, which are complementary to parts of the sequencing adapter and are added during the subsequent barcoding-PCR. All reactions were carried out following the instructions from the ‘Nextera^®^XT DNA Library Prep Reference Guide’ (Document #15031942 v01). Following amplification and DNA clean-up, DNA concentrations of the sequencing libraries were measured as recommended in the reference guide (Sect. 2.3) and samples were equimolarised to 4 ng of DNA. The concentration of the sequencing library pool from Sect. 2.6 was also measured using the TECAN infinite F200PRO fluorescence plate reader. The sequencing library pool was then diluted to a 2 nM library, resulting in a loading concentration of 10 pM (’Protocol A’ of the ‘Illumina^®^MiSeq System Denature and Dilute Libraries Guide’, Document # 15039740 v01 from January 2016). An additional step was incorporated into the denaturation step: Directly after incubation at room temperature of the 2 nM library in 0.2 N NaOH, the tube was incubated at 95 ^◦^C for 2 min. In a final step, before loading the final sequencing library pool onto the reagent cartridge, the library pool was spiked with PhiX control of *≥* 5%.

Assignment of reads to bacterial taxa and functions followed the subsequent steps. Step 1 was installation of MEGAN6 - Metagenome Analyzer^47^. Step 2 was download and extraction of the NCBI-NR database of non-redundant protein sequences (‘https://ftp.ncbi.nlm.nih.gov/blast/db/FASTA/nr.gz’). Step 3 was download and extraction of the NCBI taxdump file. Step 4 was download of NCBI accession files. Step 5 was creating a database for sequence comparison using DIAMOND^48^. Step 6 was merging and quality filtering of paired-end reads using PEAR^49^. Step 7 was conversion of FASTQ to FASTA using SED^50^. Step 8 was read alignment with DIAMOND BLASTX against a subset of the NCBI-NR database that only contained bacterial sequences (parameter: --taxonlist 2). Step 9 was meganizing the DIAMOND alignment archive files (.daa) and functional binning by mapping the NCBI database accessions of sequencing reads to KEGG Orthologs (KOs) of KEGG (acc2kegg-Dec2017X1-ue.abin) functional classifications^51,47^. KOs are functional orthologs, meaning they represent genes or proteins that perform the same function across different species. Each entry is assigned a unique identifier called a K number (e.g., K00131 for glyceraldehyde-3-phosphate dehydrogenase). Step 10 was importing the resulting files into MEGAN6 for further investigation and exporting to biom format for additional analyses in R^45^.

### Data analyses

#### Moose diet, detoxification investment and nutritional status

We quantified regional differences in dietary composition by modelling the proportional contribution of each plant group as a function of region (east vs. west) using beta regression^52,53^. For each plant variable, percentages were mapped to the open unit interval via the Smithson-Verkuilen transformation, to accommodate zeros and ones, and then fitted with *betareg* function (logit link) from the *betareg* R package^54^ as ‘y∼region’ with east as the reference. Model-based marginal means (and 95% CIs) for each region were obtained on the response scale using R package *emmeans*^55^ and reported on the percentage scale, whereby the east-west contrast was the primary estimand, with two-sided p-values derived from the asymptotic Wald test. As a sensitivity analysis robust to distributional shape, we computed Wilcoxon rank-sum tests on the untransformed percentages. We plotted results as region-specific means ± 95% CIs annotated with significance stars, the test statistic, and p-values.

Additionally, to unravel the associations among moose investment in detoxification, nutritional condition, and consumption of toxic PSCs based on proportion of balsam fir in diet, we conducted Pearson correlation analyses for proportion of balsam fir consumption vs. GA:C ratios, and proportion of balsam fir consumption vs. UN:C ratios, and GA:C ratios vs. UN:C ratios. Prior to analysis, outliers were removed using the interquartile range method^56^. Finally, we derived individual moose diet diversity using the Shannon diversity index. This metric was subsequently used as a predictor to assess the relationship between dietary diversity and microbial alpha diversity.

#### Gut microbiota diversity and composition

Explanatory variables for statistical testing were the categorical variables region (west vs. east) and age of moose (adult vs. calf), and the continuous variables balsam fir consumption, and urinary GA:C and UN:C ratios. In cases where a binary representation was required, balsam fir consumption and GA:C ratios were converted into high vs. low using the *ntile* function of the R package *dplyr*^57^. For UN:C ratios, values were considered high when above 3.5^35,36^. Unless explicitly stated otherwise, continuous variables were used for statistical testing.

##### Alpha diversity

We calculated gut microbiota alpha diversity as phylogenetic diversity (Faith’s PD)^58^ and applied a linear model to test the impact of age, region, balsam fir consumption, GA:C, UN:C, and the interactions of region with GA:C and UN:C, and interactions of balsam fir consumption with GA:C and UN:C. Model assumptions were evaluated using residual diagnostics, including assessment of linearity, homoscedasticity, normality of residuals, influential observations, and collinearity (variance inflation factors)^59^. Significance of predictors was assessed using Type II sums of squares using *Anova* function of the *car* R package^60^. As with the GA:C and UN:C ratios, we conducted Pearson correlation analyses to evaluate the relationship between Faith’s PD and the proportion of balsam fir consumption, and between Faith’s PD and dietary diversity (Shannon index).

##### Beta diversity

Multivariate community structure was modelled using redundancy analysis (RDA), implemented in the R package *vegan*^61^, with CLR-transformed genus abundances as the response matrix^62^. As for alpha diversity testing, the full model included age, region, balsam fir consumption, GA:C, UN:C, and the interactions of region with GA:C and UN:C, and the interactions of balsam fir consumption with GA:C and UN:C. Genus scores and site scores were extracted, and taxonomic annotations were merged with ordination scores to allow genus-level interpretation. Multicollinearity of constraints was assessed using variance inflation factors (VIFs), and eigenvalues of constrained axes were inspected to ensure adequate model conditioning.

To evaluate the significance of ecological predictors identified in the RDA model, we conducted a permutational multivariate analysis of variance (PERMANOVA) using the *adonis2* function of the R package *vegan*^61^ with the same formula structure. A phylogenetically informed distance matrix based on the UniFrac metric^63,64^ was computed using the R package *GuniFrac*^65^ with parameter = 0.5, which balances the sensitivity between weighted and unweighted UniFrac metric giving moderate weight to relative abundance differences while retaining sensitivity to phylogenetic structure. We used the the same distance representation for both PERMANOVA and constrained ordination. PERMANOVA tests used 999 permutations and marginal (type II-like) sums of squares. Terms with *P* < 0.05 were considered significant.

To allow simultaneous visual assessment of ordination patterns and statistical significance of ecological constraints, biplot arrows for model predictors were extracted from the RDA object and matched to PERMANOVA p-values. Significant predictors were displayed with solid lines and non-significant predictors with dotted lines. For genera, vector magnitudes were computed as √(RDA1² + RDA2²), and the top 20 genera were retained for visualization. To ensure interpretability, only genera with vector lengths > 0.1 were plotted.

##### Differential abundance analyses

Differential abundance analyses were performed at the ASV level using R package *DESeq2*^66^ to estimate variance-mean dependence to test for differential expression based on a model using the negative binomial distribution. We first conducted a series of quality-control steps on the input *phyloseq* object by removing samples with zero total read counts and ASVs with zero abundance across all retained samples. To improve numerical stability and mitigate collinearity between main effects and interactions, we centered and scaled (z-score normalization) balsam fir consumption, and GA:C and UN:C ratios. A design formula was then constructed testing the impact of region, balsam fir consumption, age, and GA:C and UN:C ratios. Size factors were estimated using the *poscounts* method to accommodate sparse microbial count data, and dispersion and model fitting followed *DESeq2*’s local trend method. We set the *fitType* parameter to *local* as it resulted in the best model fit as checked using *DESeq2*’s *plotDispEsts* function that plots the per-gene dispersion estimates together with the fitted mean-dispersion relationship. For categorical predictors, all non-reference levels were contrasted pairwise against the reference level. For continuous predictors, differential abundance was obtained using Wald statistics on the coefficient corresponding to the numeric variable. Significant ASVs were identified using Benjamini-Hochberg-adjusted p-values (false discovery rate, FDR < 0.05). For each tested predictor, a full *DESeq2* results table including taxonomy and test results was created.

##### Microbial networks and community assembly

We characterized gut microbial ecological processes and interaction structure using complementary community assembly modeling and microbial association network analyses. Analyses were conducted in R, using the packages *phyloseq*^46^ and *NetCoMi*^67^. ASVs were agglomerated to the genus level to reduce sparsity, and a global filtering step was applied prior to any group comparisons to ensure a consistent taxonomic basis for all analyses. Genera were retained if they occurred in ≥ 20% of all samples and exceeded 0.1% mean relative abundance. Taxa with zero abundance variance were removed. Samples were then stratified by ecological or physiological categories, including age, region, balsam fir consumption, detoxification status (GA:C ratio), and nutritional status (UN:C ratio). Within-group filters (≥ 5% prevalence) were applied to reduce noise from extremely rare taxa. Microbial association networks were inferred separately for each group using the *SparCC* algorithm^68^ as implemented in *NetCoMi*. Networks were analyzed using *NetCoMi*’s *netAnalyze* functions to quantify topological properties including degree, eigenvector centrality (hub identification), betweenness, and modularity, with community structure detected using the *fast-greedy* clustering algorithm. Networks were visualized using standardized layouts with node size proportional to eigenvector centrality and node color indicating cluster membership using R packages *ggplot2*^69^ and *cowplot*^70^.

To complement network analyses, we quantified ecological assembly processes using phylogenetic null-model approaches that partition beta diversity into contributions from stochastic (dispersal limitation, drift, and homogenizing dispersal) and deterministic (homogeneous selection and variable selection) processes^71^. Specifically, we applied Stegen’s null model to estimate the relative contributions of these processes based on βMNTD, βNTI and RC_bray_^71–74^ using custom code in combination with the R package *picante*^75^. Assembly processes were compared across groups defined by age, region, balsam fir consumption, detoxification status (GA:C ratio), and nutritional status (UN:C ratio) to test the impact of those factors on corresponding shifts in stochastic vs. deterministic ecological processes.

##### Gut microbiome metabolic pathways

We focused on the *Xenobiotics Biodegradation and Metabolism* and *Biosynthesis of Polyketides and Terpenoids* categories as they contain pathways involved in PSC degradation. To determine whether pathways were significantly affected by age, region, balsam fir consumption, and GA:C and UN:C ratios, we again applied *DESeq2*. Functional KEGG ortholog tables derived from shotgun metagenomics sequencing were limited in sequencing throughput and characterized by sparsity, resulting in many orthologs with very low *baseMean* values. The inclusion of multiple covariates in a multivariate *DESeq2* design inflated dispersion estimates and reduced the identifiability of individual coefficients. We thus followed established recommendations for sparse metagenomic count data and applied single-predictor *DESeq2* models to ensure stable coefficient estimation and adequate statistical power.

## Results

### Moose diet, detoxification investment and nutritional status

**Fig. 2:**
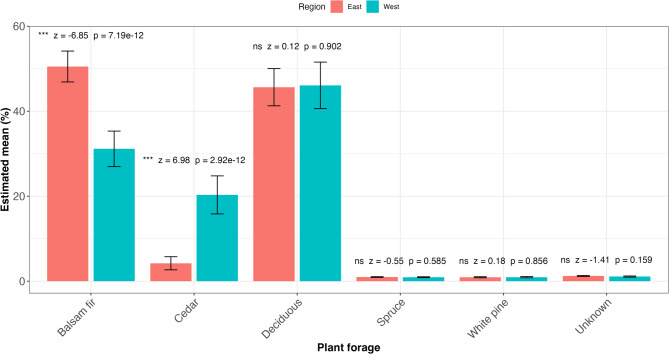
**Regional differences in moose forage.** Estimated marginal means (± SE) of the proportional contribution of major plant forage categories to moose diet in the eastern (red) and western (blue) regions of Isle Royale National Park. Significance labels represent pairwise regional contrasts derived from model-based estimates (z-statistics and associated p-values). Asterisks denote levels of statistical significance (*** *p* < 0.001; ns = not significant). Balsam fir and cedar exhibited strong regional divergence, with higher balsam fir consumption in the east and higher cedar consumption in the west. Deciduous browse and other minor forage categories (spruce, white pine, unknown) showed no detectable regional differences.

**Fig. 3:**
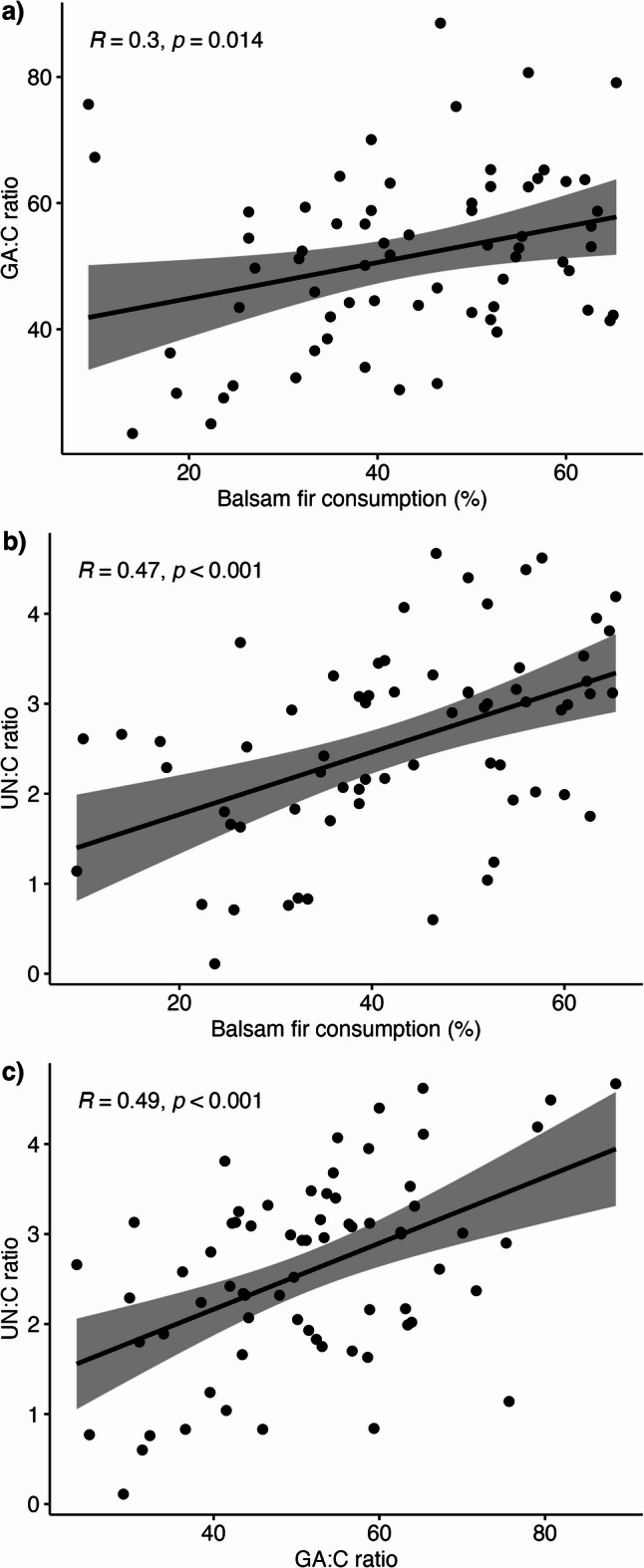
**Pearson correlation plots showing the relationships between  dietary and physiological metrics**. Positive correlation between proportion of balsam fir consumption and **a)** GA:C ratio, **b)** UN:C ratio, and between **c)** UN:C and GA:C ratios.

Dietary composition differed markedly between regions (Fig. 2). Balsam fir constituted a substantially greater proportion of the diet in the eastern compared to the western region (50.5% ± SE vs. 31.1%, β = -0.82, z = 6.9, *p* < 0.001), while cedar showed the opposite pattern, being more abundant in western diets (20.3% ± SE vs. 4.2%, z = -7.0, *p* < 0.001). For both plant taxa, the non-parametric Wilcoxon tests yielded consistent significance (*p* < 0.001). By contrast, the proportional intake of deciduous species, spruce, white pine, and unidentified plants did not differ significantly between regions. Collectively, these results indicate a pronounced east-west dietary shift driven mainly by reciprocal differences in balsam fir and cedar consumption. We also found that balsam fir consumption was positively correlated with both the GA:C ratio (t = 2.47, *R* = 0.30, *p* = 0.014; Fig. 3a) and the UN:C ratio (t = 4.3, *R* = 0.47, *p* < 0.001; Fig. 3b). Interestingly, GA:C and UN:C ratios showed a strong positive correlation (t = 2.91, *R* = 0.5, *p* < 0.001, Fig. 3c).

### Moose gut microbiota analyses

#### 16S rRNA gene sequencing

##### Microbial taxa composition

The average gut microbiota of moose from IRNP consisted of the typical herbivore community of bacterial phyla with the most abundant being Firmicutes (81.43%), Bacteroidetes (10.15%), Proteobacteria (6.27%), Verrucomicrobia (0.82%), Actinobacteria (0.69%), and Planctomycetes (0.61%) (Supplementary Table 1). When analyzing ASVs that were assigned to a genus, the most abundant genera detected in the moose were *Roseburia* (49.70%), followed by *5-7N15* (16.37%) and *Oscillospira* (6.71%) (Supplementary Table 2).

##### Alpha diversity

**Fig. 4:**
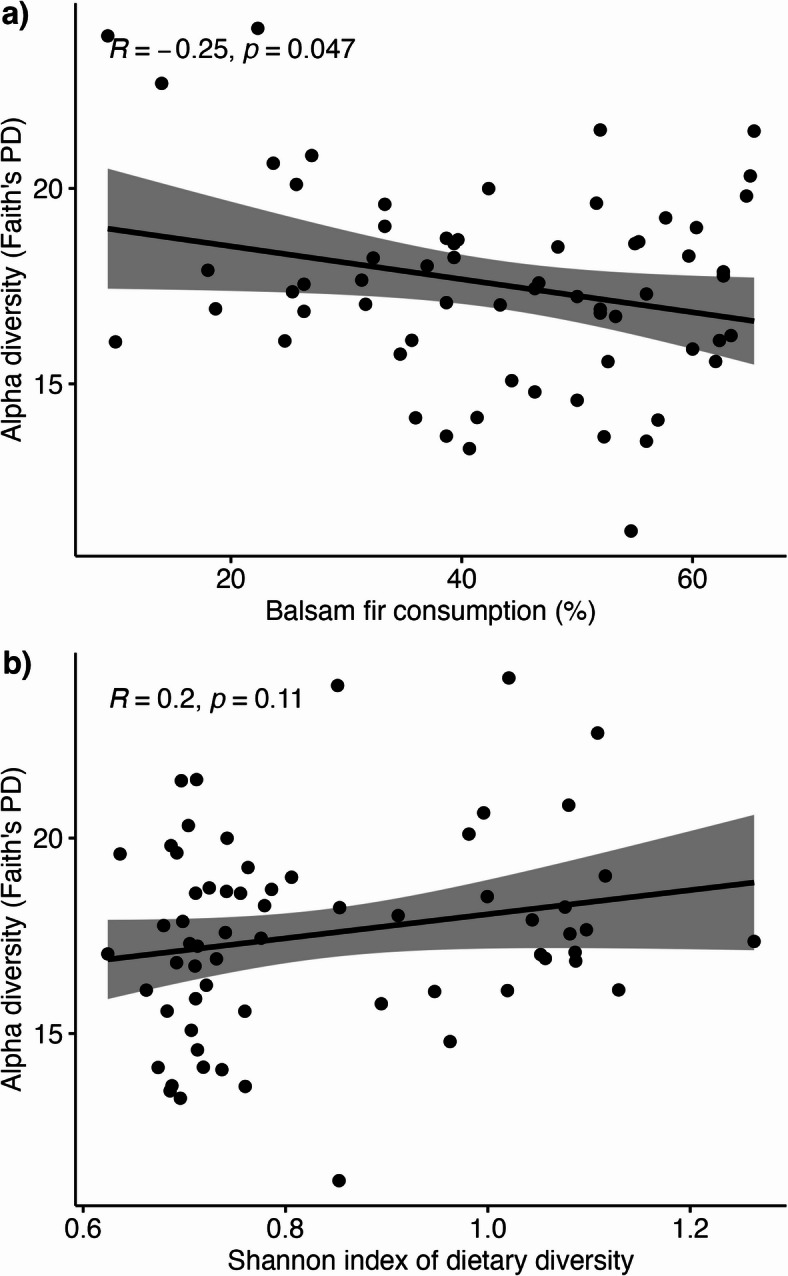
**Relationships between gut microbial phylogenetic diversity (Faith’s PD) and dietary metrics**. Pearson correlation revealed **a)** a negative relationship between PD and the proportion of balsam fir consumption and **b)** a trend for a positive relationship between Faith’s PD and dietary diversity.

**Fig. 5:**
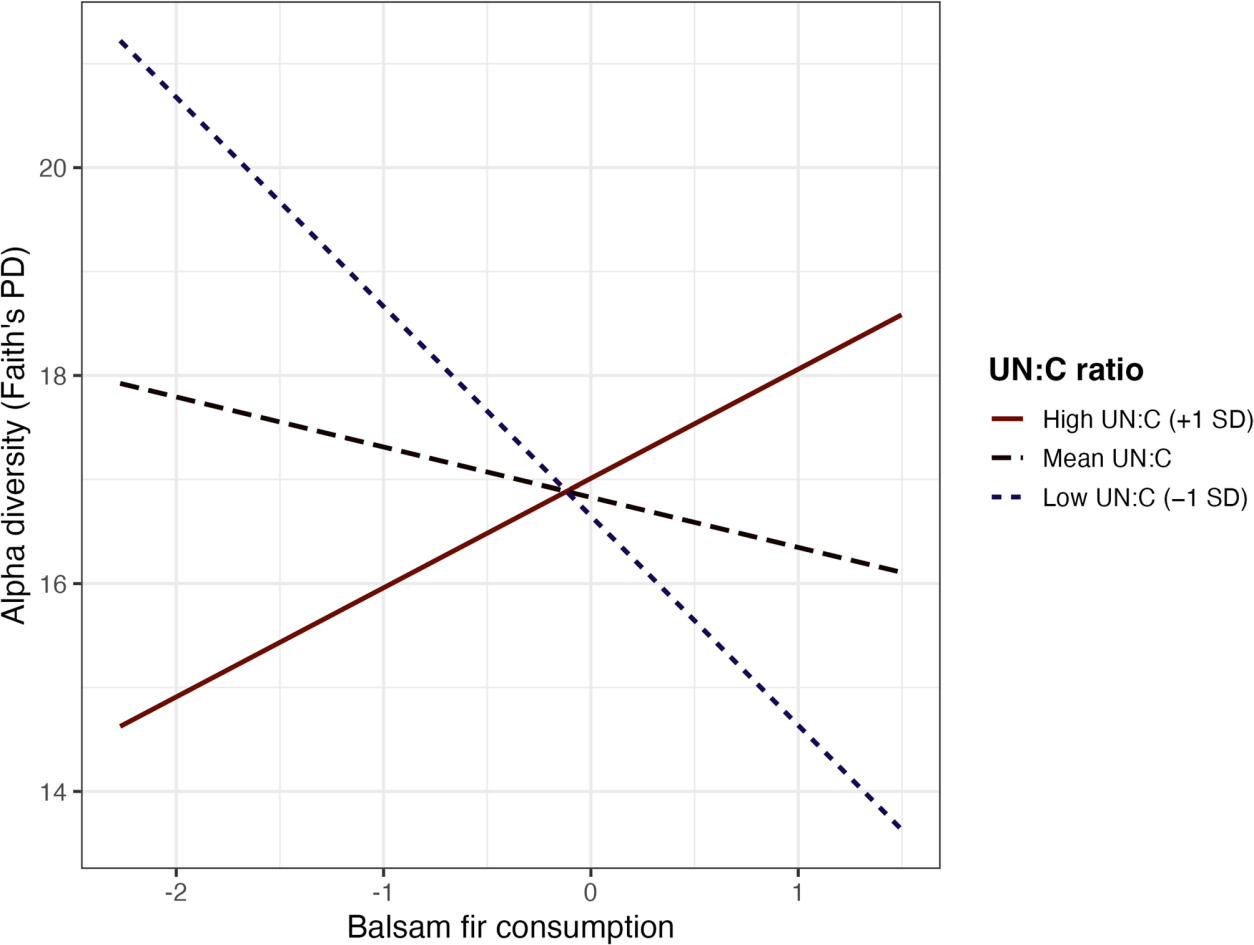
**Relationship between balsam fir consumption and gut microbial phylogenetic alpha diversity (Faith’s PD) across levels of urinary urea-nitrogen to creatinine ratio (UN:C).** Lines represent predicted Faith’s PD at high (+ 1 SD; solid brown), mean (dashed black), and low (–1 SD; dotted blue) UN:C values. The plot illustrates how the association between balsam fir consumption and gut microbial alpha diversity varies with physiological nutritional status.

Testing the effect of proportion of balsam fir consumption on gut microbial alpha diversity using a Pearson correlation revealed that the higher the proportion of balsam fir consumption, the lower the Faith’s PD (t-value = -2.02, *r* = -0.25, *p* = 0.047, Fig. 4a). A positive trend existed for the relationship between dietary diversity and gut microbial alpha diversity (Faith’s PD), although not statistically significant (t-value = 1.63, *r* = 0.20, *p* = 0.108, Fig. 4b).None of the main effects, age (F₁,₅₃ = 0.27, *p* = 0.603), region (F₁,₅₃ = 0.11, *p* = 0.742), balsam fir consumption (F₁,₅₃ = 2.23, *p* = 0.141), GA:C (F₁,₅₃ = 0.43, *p* = 0.516), or UN:C (F₁,₅₃ < 0.01, *p* = 0.996) were statistically significant in their relationship with gut microbial diversity. Among interaction terms, balsam fir consumption × UN:C was the only significant predictor of Faith’s PD (F₁,₅₃ = 11.83, *p* = 0.001). To facilitate interpretation of this interaction, we created an interaction plot (Fig. 5). All other interaction terms were non-significant. Residual diagnostics indicated that model assumptions were adequately met.

##### Beta diversity

**Fig. 6:**
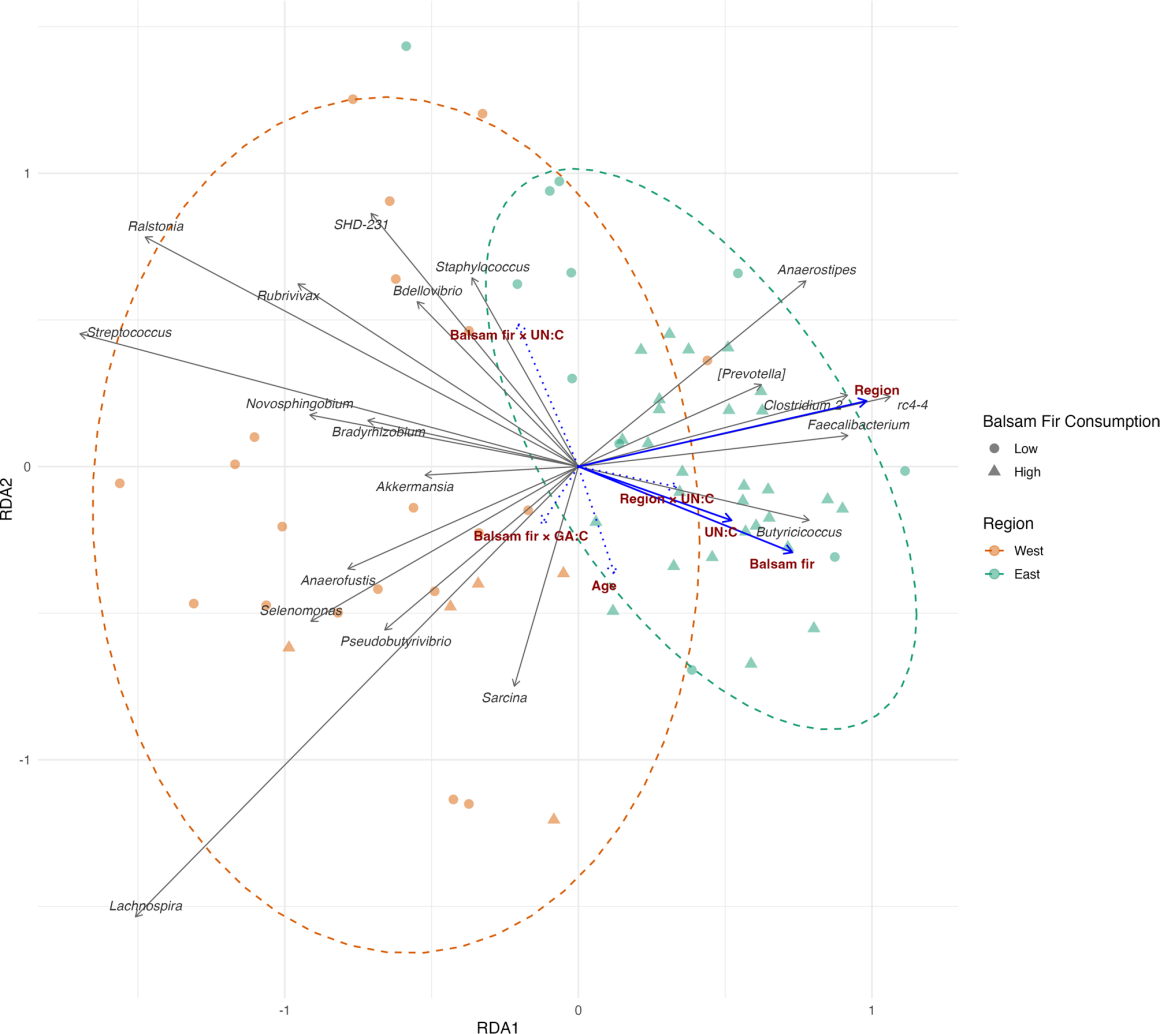
**Multivariate redundancy analysis plot** **illustrating associations between** ** moose gut microbiota** **and** **extrinsic (environmental) and intrinsic (physiological) factors.** Samples are colored by region and shaped by categorical balsam fir consumption (low vs. high), with 95% confidence ellipses for each region. Genus vectors are shown as grey arrows with italicized labels, whereas predictors are shown as blue arrows annotated with descriptive labels and significance coding based on PERMANOVA, where continuous lines are significant and dotted lines are non-significant predictors.

For beta diversity, results of the PERMANOVA revealed significant effects of region (F_1,63_ = 6.0, R^2^ = 0.08, *p* = 0.001), balsam fir consumption (F_1,63_ = 4.4, R^2^ = 0.06, *p* = 0.002), and UN:C ratio (F_1,63_ = 2.7, R^2^ = 0.04, *p* = 0.009). There was a marginal statistical significance for the interaction of balsam fir consumption and UN:C (F_1,63_ = 1.62, R^2^ = 0.02, *p* = 0.095) on beta diversity, whereas there was no effect of GA:C (F_1,63_ = 1.32, R^2^ = 0.02, *p* = 0.188). The sum of R² for significant terms was 17.6%. The RDA model explained 22.5% of the total variance in CLR-transformed gut microbial community composition (constrained inertia = 14.48). The remaining inertia was unconstrained (49.83; 77.5%) by the environmental and physiological variables included in the model. The overlay of PERMANOVA test results with the RDA plot nicely visualizes how the different environmental and physiological factors explain the separation of moose gut microbiota (Fig. 6).

##### Differential abundance analyses

Differential abundance analysis using *DESeq2* on data agglomerated on genus level identified eight genera that differed significantly with respect to moose age class (Supplementary Table 3), among which *Clostridium* (log₂FC = -27.6, baseMean = 4.51, p-adjusted < 0.001) and *Mycoplasma* (log₂FC = -18.5, baseMean = 1.06, p-adjusted < 0.001) exhibited the largest fold-change being more abundant in adults. Fifteen genera were associated with region (Supplementary Table 4) with several genera displaying large fold-changes, including *Nocardioides* (log₂FC = 15.6, baseMean = 0.14, p-adjusted = 0.002 ) and *Brevundimonas* (log₂FC = 14.7, baseMean = 0.19, *p* < 0.001), which ranked among the strongest habitat-associated responses being more abundant in eastern compared to western moose. Six genera were significantly associated with balsam fir consumption (Supplementary Table 5). The largest effects within this predictor included *Actinomycetospora* (log₂FC = 16.6, baseMean = 0.12, p-adjusted < 0.001) and *Nocardioides* (log₂FC = 11.6, baseMean = 0.14, p-adjusted < 0.001) being more abundant when balsam fir consumption was high. Importantly, *Roseburia* was also identified as significantly associated with increasing balsam fir consumption (log₂FC = 0.24, baseMean = 11,046, p-adjusted = 0.037), representing one of the genera with the highest abundance detected across all significant results. Detoxification status (GA:C) was associated with five genera (Supplementary Table 6), among which *Brevundimonas* displayed the largest absolute fold change (log₂FC = -14.5, baseMean = 0.19, p-adjusted < 0.001) being less abundant when GA:C was higher, followed by *Kocuria* (log₂FC = 7.19, baseMean = 0.08, p-adjusted < 0.001) being more abundant when GA:C was higher. Three genera were significantly associated with nutritional status (UN:C) (Supplementary Table 7). The largest effects were observed for *Methyloversatilis* (log₂FC = -6.82, baseMean = 0.22, p-adjusted = 0.013) and *Nocardioides* (log₂FC = -6.62, baseMean = 0.14, p-adjusted = 0.013), being less abundant when UN:C was higher.

##### Network and community analysis

**Fig. 7:**
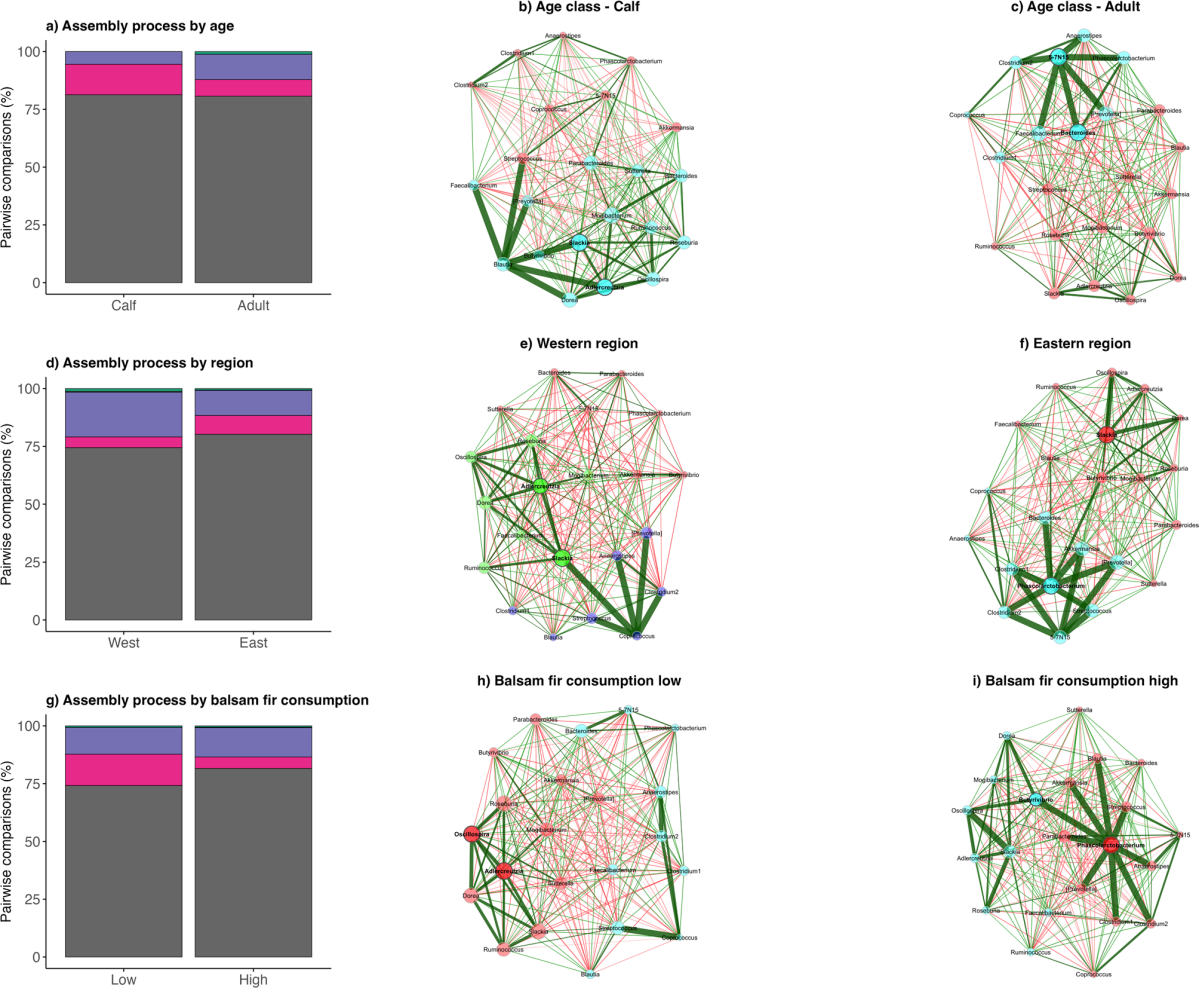
**Microbial community assembly processes and co-occurrence network structure across extrinsic (environmental: region) and intrinsic (moose: age and balsam fir consumption) gradients in moose.** Stacked bar plots (left panels) summarize the proportional contribution of community assembly processes to pairwise community turnover for **a)** age class (calf vs. adult), **d)** region (west vs. east), and **g)** balsam fir consumption (low vs. high). Assembly processes are color-coded as follows: dispersal limitation (purple), drift/undominated processes (grey), homogeneous selection (orange), homogenizing dispersal (pink) and variable selection (green). Corresponding co-occurrence networks (central and right panels) display genus-level gut microbial associations for **b)** calves and **c)** adults; **e)** west and **f)** eastern regions; and **h)** low and **i)** high balsam fir consumption groups. Nodes represent gut microbial genera and are scaled by eigenvector centrality, while edges represent significant pairwise associations (green = positive; red = negative).

**Fig. 8:**
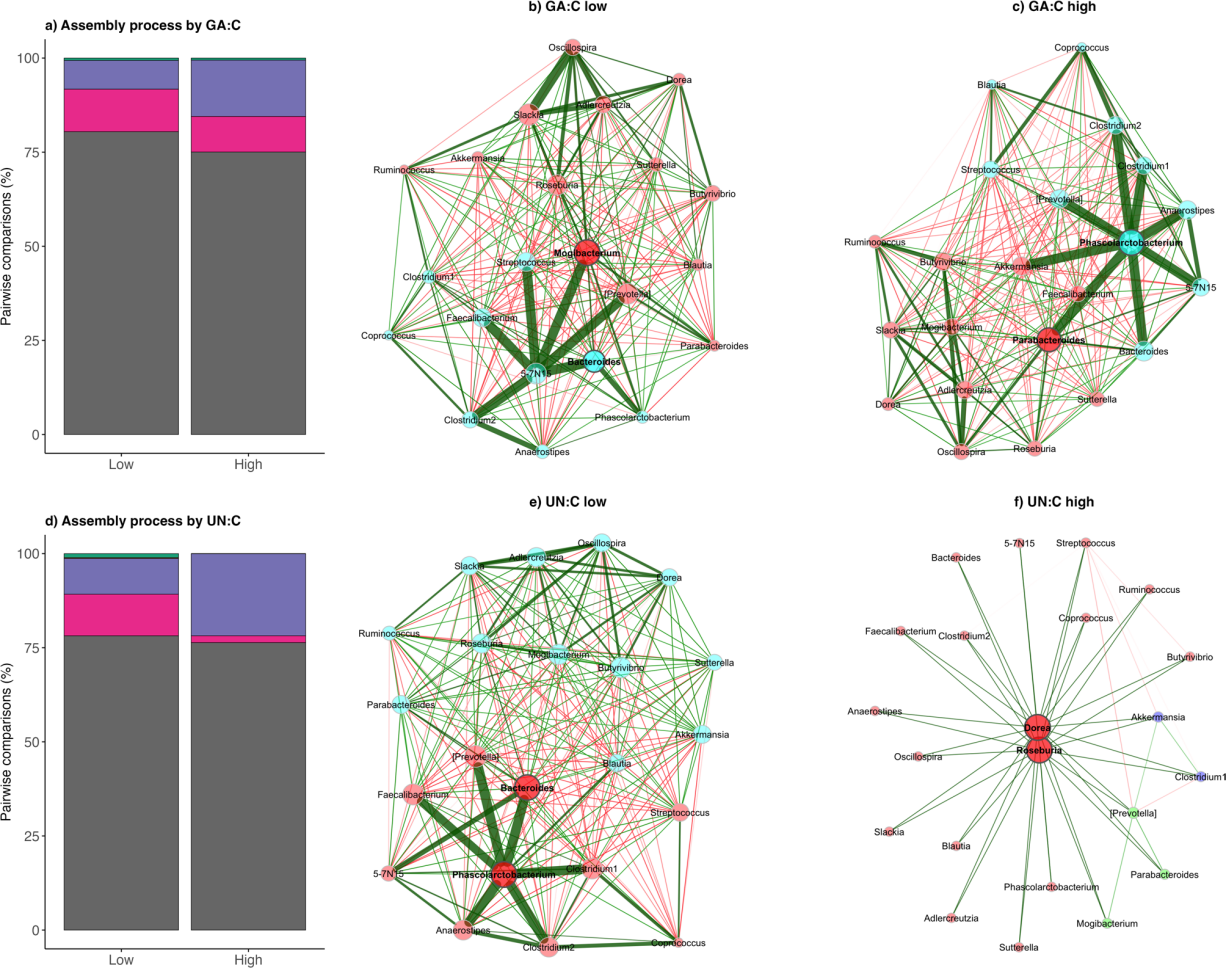
**Microbial community assembly processes and microbial co-occurrence network structure across intrinsic physiological gradients in moose**. Stacked bar plots (left panels) display the proportional contribution of community assembly processes to pairwise community turnover for **a)** glucuronic acid-to-creatinine (GA:C) categories (low vs. high) and **d)** urea nitrogen-to-creatinine (UN:C) categories (low vs. high). Assembly processes are color-coded as follows: dispersal limitation (purple), drift/undominated processes (grey), homogeneous selection (orange), homogenizing dispersal (pink) and variable selection (green). Corresponding co-occurrence networks (central and right panels) show genus-level gut microbial associations inferred for **b)** low and **c)** high GA:C groups, and for **e)** low and **f)** high UN:C groups. Nodes represent gut microbial genera and are scaled according to eigenvector centrality, and edges denote significant associations (green = positive; red = negative).

Across all factors examined, gut microbial community assembly was consistently dominated by drift or undominated processes, which accounted for approximately 74–82% of all pairwise comparisons, indicating a strong and pervasive influence of stochasticity across age classes, regions, and balsam fir consumption (Fig. 7), and GA:C and UN:C ratios (Fig. 8). In contrast, deterministic selection processes were uniformly rare, with homogeneous and variable selection together contributing ≤ 1–2% in all comparisons, underscoring their limited role in structuring communities under the conditions examined. Within this shared stochastic framework, the most notable differences among factors were expressed through dispersal-related processes. Age classes exhibited largely comparable assembly profiles, with drift or undominated processes occurring at similar frequencies in adults (80.7%) and calves (81.3%). The primary age-related difference was a higher contribution of homogenizing dispersal in calves (13.2%) compared to adults (7.2%), while variable selection was detected only in adults (1.1%). Regional comparisons revealed clearer contrasts, with dispersal limitation occurring more frequently in the western (19.4%) than in the eastern region (10.9%), whereas homogenizing dispersal was less prevalent in the west (4.6%) relative to the east (8.1%). Balsam fir consumption, GA:C and UN:C ratios showed the strongest shifts in dispersal dynamics. Along the balsam fir consumption gradient, low consumption was associated with a substantially higher contribution of homogenizing dispersal (13.5%) compared to high consumption (4.9%), while drift or undominated processes remained dominant in both groups (74.2–81.6%). Similarly, high GA:C ratios were characterized by increased dispersal limitation (15.0%) relative to low GA:C ratios (7.7%). A comparable pattern was observed for nutritional status, where high UN:C ratios exhibited markedly higher dispersal limitation (21.8%) and minimal homogenizing dispersal (1.8%), whereas low UN:C ratios showed lower dispersal limitation (9.5%) and substantially greater homogenizing dispersal (11.0%).

Networks stratified by moose age, region, and balsam fir consumption were all densely connected, but differed in the prominence and distribution of highly central nodes, with some groups (e.g. adults, eastern region, high balsam fir consumption) exhibiting more concentrated hub structures and thicker positive edges around a limited subset of taxa, whereas others (calves, western region, low balsam fir consumption) showed a more even distribution of centrality across the network (Fig. 7). Physiological predictors revealed even stronger topological contrasts with low GA:C ratio (lower detoxification) and low UN:C ratio (higher nutritional status) networks forming dense, mesh-like architectures with many interlinked hubs, whereas especially the high UN:C ratio (poorer nutritional status) network collapsed into a star-like configuration dominated by two highly central nodes connected to numerous peripheral taxa and relatively few links among non-hub nodes (Fig. 8).

#### Shotgun metagenomic sequencing

##### KEGG functional profiling

Read alignment using DIAMOND BLASTX against a subset of the NCBI-NR database containing only bacterial sequences resulted in 1,246,723 matches. Subsequent functional binning by mapping the NCBI database accessions to KEGG functional classification identifiers resulted in 528,529 matches to KEGG orthologs (KOs).

**Fig. 9:**
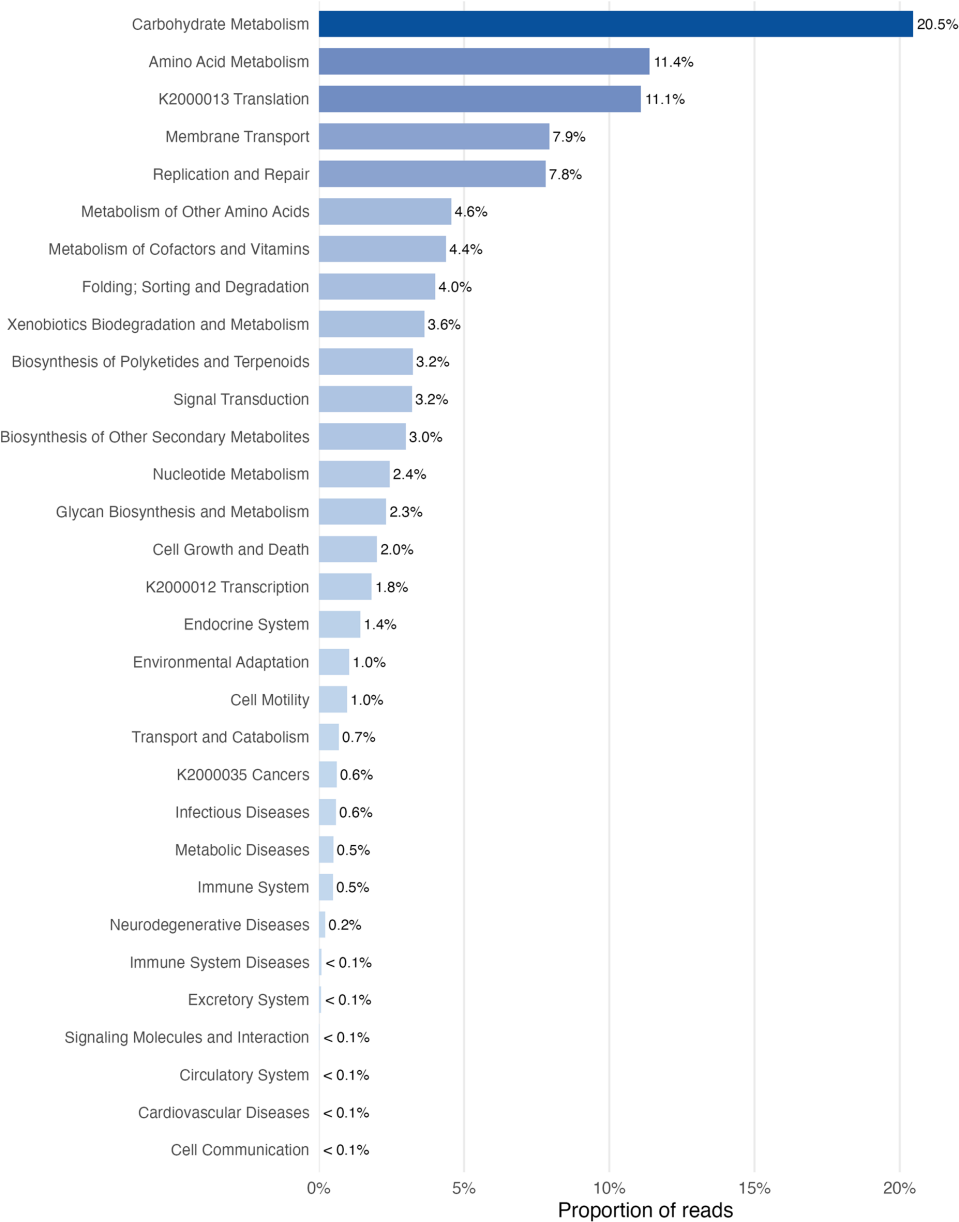
**Proportions of KEGG functional pathway categories across metagenomic data  derived  from moose fecal samples.** Horizontal bars represent the proportional contribution of each pathway, ordered from highest to lowest abundance.

Functional profiling of KEGG pathway categories revealed that gut microbial communities were dominated by metabolic processes (Fig. 9). *Carbohydrate Metabolism* constituted the largest proportion of predicted functional potential (20.5%), followed by *Amino Acid Metabolism* (11.4%) and *KO20013 Translation* (11.1%). Additional functions with notable representation included *Membrane Transport* (7.9%) and *Replication and Repair* (7.8%). Pathways associated with nutrient processing and cellular maintenance, such as *Metabolism of Other Amino Acids* (4.6%), *Metabolism of Cofactors and Vitamins* (4.4%), and *Folding*,* Sorting and Degradation* (4.0%) were also moderately abundant. Xenobiotic-related pathways, including *Xenobiotics Biodegradation and Metabolism* (3.6%) and *Biosynthesis of Polyketides and Terpenoids* (3.2%), contributed smaller but relevant proportions. All remaining pathway categories individually accounted for ≤ 3% of total predicted reads, with several (e.g., *Cell Communication*, *Cardiovascular System*, *Circulatory System*) representing < 0.1%.

**Fig. 10:**
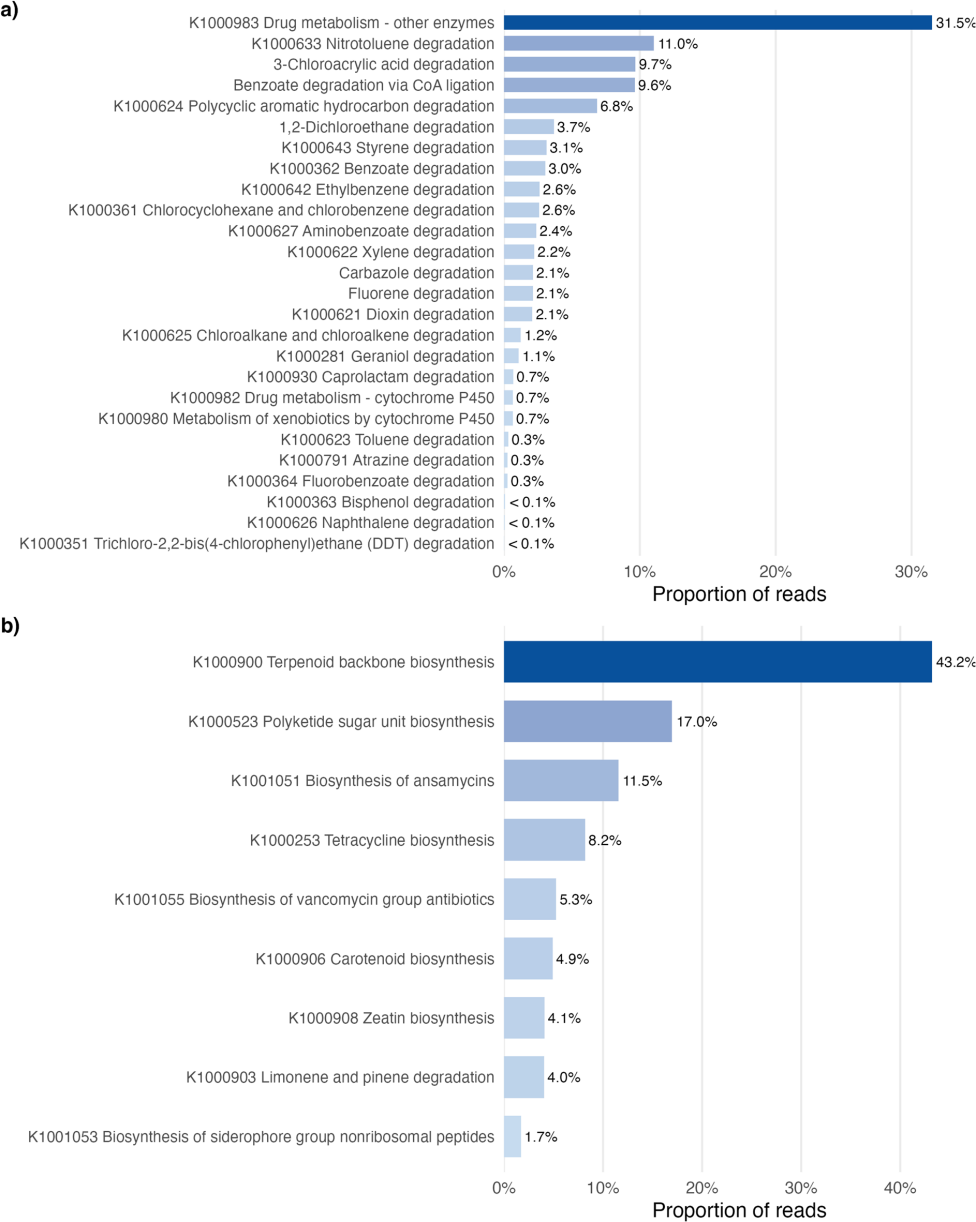
**Proportion of KEGG functional pathways** **across metagenomic data derived from moose fecal samples.** Horizontal bars represent the proportion of reads assigned to a pathway within the **a)** *Xenobiotics Biodegradation and Metabolism* or **b)** *Biosynthesis of Polyketides and Terpenoids* pathway categories across moose fecal metagenomic samples.

Within the KEGG functional category *Xenobiotics Biodegradation and Metabolism* (Fig. 10a), microbial communities were overwhelmingly dominated by *Drug metabolism- other enzymes*, which accounted for 31.5%. Additional pathways with substantial representation included *Nitrotoluene degradation* (11.0%) and *Benzoate degradation* (9.6%), followed by *Polycyclic aromatic hydrocarbon degradation* (6.8%). Several other aromatic compound degradation pathways were present at lower but detectable levels, such as *1*,*2-dichloroethane degradation* (3.7%), *Styrene degradation* (3.1%) or *Benzoate degradation* (3.0%). Most remaining xenobiotic pathways each contributed < 3% of reads.

In the *Biosynthesis of Polyketides and Terpenoids* category (Fig. 10b), functional potential was dominated by *Terpenoid backbone biosynthesis*, comprising 43.2% of all reads assigned to this category. *Polyketide sugar unit biosynthesis* (17.0%) and *Biosynthesis of ansamycins* (11.5%) were also prominently represented. Other pathways exhibited moderate contributions, including *Tetracycline biosynthesis* (8.2%), *Biosynthesis of vancomycin group antibiotics* (5.3%), and *Carotenoid biosynthesis* (4.4%). Functions associated with *Zeatin biosynthesis*,* Limonene and pinene degradation*, and *Biosynthesis of siderophore group non-ribosomal peptides* each accounted for < 5% of reads.

##### Differential abundance analyses

**Fig. 11:**
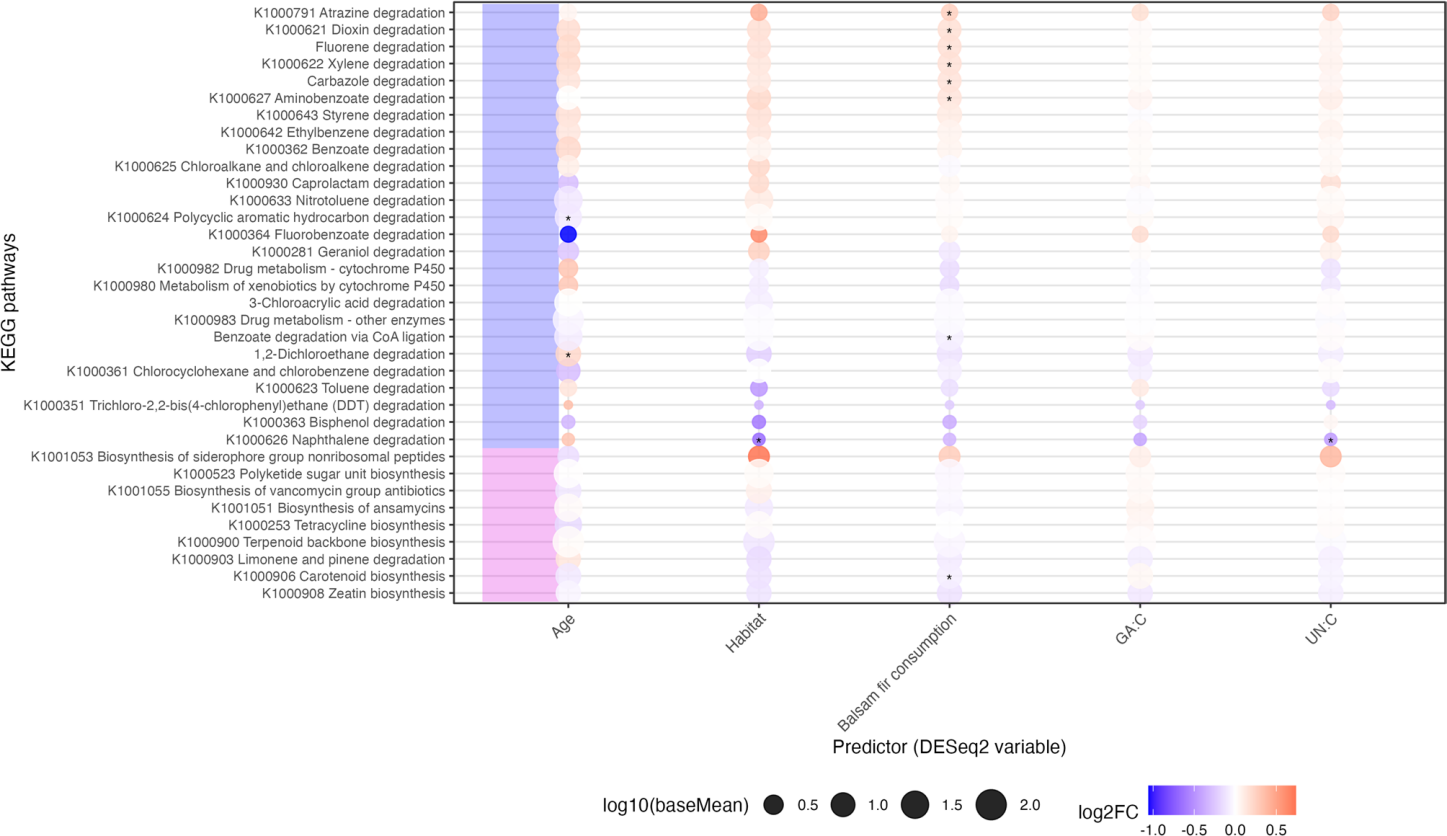
**Differential abundance of KEGG pathways across extrinsic (environmental) and intrinsic (physiological) predictors.** Bubble plot displaying the log2-fold-change (log2FC) of each pathway for the predictors age, region, balsam fir consumption, GA:C, and UN:C. Each bubble represents a pathway-predictor association, with bubble size proportional to log10(baseMean), reflecting overall pathway abundance, and bubble color denoting the direction and magnitude of log2FC (blue = decreased abundance; red = increased abundance). Stars indicate significant p-value before correction of multiple testing (all p-adjusted were non-significant).

A total of twelve KEGG pathways exhibited nominal differences in abundance across environmental and physiological predictors (*p* < 0.05), although none remained significant after false-discovery rate correction (Fig. 11, Supplementary Tables 8–12). Age effects were limited to the pathways *Chlorocyclohexane and chlorobenzene degradation (K1000361)* and *Fluorobenzoate degradation (K1000364)*, both of which showed reduced abundance in adults relative to calves. Regional differences were restricted to the *Biosynthesis of siderophore group nonribosomal peptides (K1001053)* pathway, which was more abundant in the eastern region. Balsam fir consumption was associated with the largest number of pathways and showed a consistent pattern in which several xenobiotic and aromatic compound degradation pathways increased with higher balsam fir consumption, including *Dioxin degradation (K1000621)*, *Xylene degradation (K1000622)*, *Styrene degradation (K1000643)*, *Aminobenzoate degradation (K1000627)*, *Fluorene degradation*, and *Carbazole degradation*. In contrast, *Zeatin biosynthesis (K1000908)* and *1*,*2-Dichloroethane degradation* decreased with balsam fir consumption. UN:C ratio showed a nominal positive association with *Biosynthesis of siderophore group nonribosomal peptides (K1001053)*, the only pathway linked to more than one predictor. Although most test results did not reach nominal significance, several pathways displayed consistent directional patterns across predictors. In particular, the same set of xenobiotic and aromatic degradation pathways that increased with high balsam fir consumption also tended to show higher abundance in samples from the eastern region, suggesting a shared directional response despite limited statistical support.

## Discussion

We examined how dietary exposure to plant secondary compounds, host physiological state, and gut microbial communities interacted in free-ranging moose on IRNP. By integrating fecal diet composition, urinary biomarkers of detoxification and nutritional status, and 16S rRNA gene and shotgun metagenomic sequencing, we assessed how a PSC-rich winter diet influenced both host metabolic investment and gut microbial structure to cope with this extreme situation.

We hypothesized that balsam fir consumption would be positively associated with moose investment in detoxification. Our results support this hypothesis, because moose in the eastern region of IRNP consumed a higher proportion of balsam fir than those in the western region^32 and^ the amount of balsam fir consumed was positively correlated with both GA:C and UN:C ratios. This means that moose feeding on larger amounts of balsam fir had a higher metabolic investment in detoxification and were more nutritionally restricted. In addition, GA:C and UN:C ratios were positively correlated which suggests that the behavioral and physiological processes involved in the ingestion, absorption, and detoxification of PSCs, particularly through the glucuronidation pathway, may negatively impact moose nutritional status. In fact, moose individuals that had UN:C ratios > 3.5 lived predominately in the eastern region of IRNP and consumed more balsam fir. Balsam fir in the eastern region is known to have less protein than the ones in the western region and consuming high concentrations of PSCs whilst on a low-protein diet can cause a negative nitrogen balance^76^. These patterns are consistent with earlier findings for this population^32^ and with independent annual patterns reported previously^38^. The link between PSC intake and GA are consistent with findings across vertebrate taxa. White-tailed deer (*Odocoileus virginianus*) fed on a diet consisting of 55% of balsam fir had GA:C ratios almost threefold higher compared to a diet based on deciduous plants^77^. Mule deer (*Odocoileus hemionus*) and white-tailed deer increased GA excretion with increasing intake of PSCs^78^. In addition, ruffed grouse (*Bonasa umbellus*) assimilated less energy, excreted more GA and ornithine, and reduced body mass when fed a PSC-rich diet^79,80^. Both the type of PSC and the herbivore’s ecological experience with PSC may influence reliance on GA and nutritional costs of PSC intake. For example, intake of monoterpenes resulted in higher GA excretion than the intake of tannins in deer^78^ In addition, feeding experiments carried out on specialist (*Neotoma stephensi*) and generalist (*N. albigula*) woodrats, revealed that specialist were less energetically impacted by high PSC in juniper foliage (*Juniperus monosperma*) ) and excreted less GA relative to PSC intake than generalists^81^. Interestingly, research has shown that in addition to the herbivore physiology itself, gut microbiota play an important role in detoxification of PSC^82^ and therefore may influence reliance on GA by the host.

Inspired by those pioneer studies on the role of gut microbiota in detoxification of PSC in herbivores, we further hypothesized that differences in diet between moose from the eastern and western region of IRNP should affect gut microbial diversity and composition, with an increase of bacterial taxa associated with PSC detoxification associated with a greater intake of balsam fir. We found that the phylogenetic diversity of individual moose gut microbiota was negatively correlated with the proportion of balsam fir consumption and positively, albeit not significantly, with dietary diversity. This pattern aligns with previous studies showing that a chemically defended diet can shape gut microbial composition and even reduce microbial diversity^26,28,83,84^. The significant stress and selective pressure on moose gut microbial communities to adopt bacterial taxa with an increased capacity to detoxify PSC may have led to the observed decrease in alpha diversity. Thus, the lower gut microbial alpha diversity in moose from the eastern region of IRNP is most likely due to a shift in the gut microbiota community driven by a less diverse and PSC-enriched diet. Results indicate that the moose gut microbiota may shift towards a more specialized bacterial community that is beneficial for moose when counteracting PSC.  One potential mechanism underlying shifts in moose gut microbiota composition is the broad-spectrum antimicrobial activity of balsam fir tannins.^85^.

Interestingly, the variation in phylogenetic diversity (Faith’s PD) was not explained by individual factors such as age, region, or the main effects of diet and physiological indicators when considered independently. Instead, the strong and highly significant interaction between balsam fir consumption and UN:C ratios indicates that the relationship between diet composition and gut microbial diversity is fundamentally conditional on the nutritional status of individual moose (Fig. 5). Moose in good nutritional condition maintained or even increased gut microbial alpha diversity when consuming more balsam fir, while nutritionally limited individuals (UN:C > 3.5) experienced steep declines in diversity as fir consumption increased. These patterns highlight that the gut microbiota consequences of consuming PSC-rich forage are not uniform across individuals but are modulated by physiological state. Nutritional status appears to determine whether balsam fir consumption aligns with gut microbiota maintenance or disruption. The absence of interactions involving GA:C suggests that detoxification activity may be less influential than nutritional status in shaping gut microbiota responses to balsam fir consumption within this dataset. Although further work is needed to partition the relative contributions of toxin load and nutrient balance in moose, studies on other herbivores have shown gut microbiota changes in response to nutritional stress^86^. Consistent with findings in howler monkeys^87^, our results suggest the existence of a threshold in plant secondary compound (PSC) intake beyond which the moose gut microbiota shifts in a manner that diminishes its nutritional contributions to the host.

In contrast to phylogenetic alpha diversity, beta diversity analyses revealed the impact of region, age, balsam fir consumption, and UN:C ratio on gut microbial community composition, whereas GA:C ratio did not. As with Faith’s PD, there was an interaction of balsam fir consumption and UN:C. The strongest driver in explaining beta diversity differences was region, indicating that in addition to the factors investigated in this study, other factors specific to the western or eastern region of IRNP contributed to shaping gut microbial communities. The fact that there was a dispersal limitation between the two moose populations contributed further to gut microbiota community composition differences^32^. Obviously, regional belonging is an umbrella variable behind which many other drivers could potentially act upon gut microbiota community differences like social complexity^88^, heritability^89^, or an almost infinite range of subtle environmental differences as was shown for an island bird population where gut microbiota communities exhibited high-resolution spatial variation over fine scales^90^.

On the bacterial genus level, the positive association between balsam fir consumption and *Roseburia*, identified through differential abundance testing, is particularly noteworthy because *Roseburia* was among the most abundant genera in moose and is a known butyrate-producer. Butyrate-producing bacteria have been linked to the regulation of hepatic detoxification genes (Lucas et al. 2022) and can influence gut epithelial integrity and host xenobiotic metabolism^91,92^.

The assembly analyses revealed that stochastic processes structured the moose gut microbiota across demographic, spatial, dietary, and physiological gradients, with drift or undominated processes accounting for approximately 74–82% of pairwise comparisons. Deterministic selection was consistently rare across all factors with variable selection and homogeneous selection playing a limited role in shaping microbial community composition in this system. Variation among factors was primarily expressed through shifts in dispersal-related processes. Age-related differences were modest, with broadly similar assembly profiles in adults and calves, although calves exhibited a higher contribution of homogenizing dispersal, suggesting greater gut microbiota similarity among juveniles^93^. Regional effects were more pronounced, with increased dispersal limitation in the western region and higher homogenizing dispersal in the eastern region, suggesting that gut microbial exchange was higher among moose of the east. Balsam fir consumption and physiological gradients showed the clearest divergence in assembly dynamics. Low balsam fir consumption and low GA:C ratios were associated with higher contributions of homogenizing dispersal, whereas high balsam fir intake and elevated GA:C ratios corresponded to increased dispersal limitation. Moose with poor nutritional status (high UN:C ratios) exhibited a particularly strong pattern with markedly greater dispersal limitation and minimal homogenizing dispersal, while individuals with better nutritional status (low UN:C ratios) displayed substantially greater microbial exchange. Collectively, these results indicate that while stochastic processes dominate the moose gut microbiome assembly, ecological, dietary, and physiological gradients modulated community structure primarily through shifts in dispersal dynamics. Rather than increasing deterministic selection, poorer nutritional and detoxification states were associated with increased dispersal limitation and greater gut microbiota individuality, highlighting dispersal as a key mechanism linking moose condition to their gut microbiota assembly.

Microbial network structure differed markedly across dietary and physiological gradients, despite overall community assembly being dominated by drift or undominated processes. Low PSC exposure and more favorable nutritional status of moose produced densely connected networks with many interacting hubs such as *Faecalibacterium*,* Bacteroides*,* Coprococcus*,* Prevotella*,* Roseburia and Ruminococcus*. Moose with high PSC exposure displayed a restructured network where bacterial taxa that were distantly associated with each other suddenly showed strong connections forming a strong hub with the central taxon *Phascolarctobacterium*. Some of the associated bacterial taxa are known to be involved in metabolism of aromatic plant compounds^94^ or acting as important butyrate-producers^95,96^. The biggest difference was observed between low and high UN:C ratio networks where under strong nutritional restrictions the network experienced a strong reorganization with central hubs formed by *Roseburia* and *Dorea*, another genus of the Firmicutes capable of producing SCFA^97^. As in moose, PSC-associated network reorganizations have also been observed in woodrats^98^ and black howlers monkeys^87^. Taken together, these findings indicate that detoxification is not reflected merely in gut microbiota diversity shifts, but in a broader rewiring of gut microbial interactions. This supports the view that the gut microbiome contributes functionally to PSC tolerance in wild herbivores, not only through the presence of bacterial taxa capable of detoxifying PSCs, but through the emergence of PSC-adapted microbial network architectures that may enhance metabolic resilience.

We further hypothesized that the increased need for PSC detoxification associated with a diet rich in PSCs would be reflected in KEGG functional pathways of the moose gut microbiome. Indeed, shotgun metagenomic sequencing revealed that moose gut microbiota carried genes corresponding to pathways involved in the biodegradation of xenobiotics such as the prominent *Benzoate degradation via CoA ligation* and *Benzoate degradation* pathway. Many toxic compounds, such as polycyclic aromatic hydrocarbons or phenolics, are typically degraded to benzoate, which then enters the benzoate degradation pathway for further degradation to acetyl-CoA, which can be consumed in the citrate cycle. The importance of bacteria involved in benzoate degradation has also been demonstrated in other herbivore species that feed on plants containing potentially toxic PSCs^99^. A striking contrast was the relatively low representation of the *Limonene and pinene degradation* pathway, which accounted for less than 5%, despite the high concentrations of β-pinene and R-limonene typically found in balsam fir^100^. This finding suggests that gut microbial activity may be directed toward other PSCs present in balsam fir foliage. Compounds such as camphene, Δ3 carene, α terpineol, piperitone, bornyl acetate, thymol, longifolene, and β bisabolene typically present in balsam fir^101^ may have engaged distinct microbial degradation pathways.

When testing for differentially abundant KEGG functional pathways, we did not identify significant differences in abundance of xenobiotic pathways after correction for multiple testing. This is in line with a study on woodrats where despite gut microbiome community differences, no difference in the functional profiles between PSC and non-PSC feeding woodrats could be observed^102^. Nevertheless, a trend was detected with several pathways such as *Dioxin degradation (K1000621)*, *Xylene degradation (K1000622)* or *Styrene degradation (K1000643)* being more abundant when balsam fir consumption by moose was higher.

Overall, our results indicate that moose responded to the chemical and nutritional challenges of a PSC-rich winter diet through a combination of host physiology and gut microbial functions. Balsam fir consumption imposed clear physiological costs on moose and simultaneously restructured the gut microbiome at multiple levels, including diversity, taxonomic composition, interaction networks, and functional potential. Although we found evidence that the moose gut microbiome carried genes involved in PSC detoxification and that some bacterial groups responded to balsam fir consumption, there was no indication that microbial detoxification pathways were upregulated in a manner that mirrored physiological detoxification responses of moose. It seems that the combined detoxification capacities of moose and their gut microbiomes might be sufficient to deal with the amount of ingested PSC independent of whether balsam fir intake was lower or higher. It appears that the additional support of the gut microbiome under a high balsam fir consumption regime is to maintain fermentative function and network stability under chemically and nutritionally challenging conditions. Finally, the presence of several bacterial taxa capable of producing short-chain fatty acids such as butyrate reveals an important interaction by which bacteria can boost host metabolic detoxification processes^103^. Altogether, the detoxification capabilities of moose, their gut microbiota, and more importantly, their interactions, enable moose to persist on a winter diet that is both energetically limited and chemically demanding in the extreme ecological context of IRNP.

## Conclusion

This study demonstrated that a winter diet dominated by balsam fir imposes measurable detoxification and nutritional demands on moose and that these pressures are reflected in distinct shifts in gut microbial diversity, community composition, and network structure. Moose gut microbiota exhibited microbial pathways with the capacity to degrade aromatic and terpenoid PSCs. Balsam fir consumption was associated with changes in the prominence of key fermentative taxa, including butyrate-producing genera such as *Roseburia* and taxa involved in intermediary metabolism such as *Phascolarctobacterium*. These gut microbial shifts, together with host detoxification responses, indicate that PSC tolerance emerges from an integrated interaction between host physiology and the gut microbiome. Collectively, these findings provide new insight into how large herbivores persist on PSC-rich winter forage and underscore the importance of considering both host and gut microbial processes when evaluating herbivore dietary ecology.

## Supplementary Information

Below is the link to the electronic supplementary material.

Supplementary Material 1

Supplementary Material 2

Supplementary Material 3

Supplementary Material 4

Supplementary Material 5

Supplementary Material 6

Supplementary Material 7

Supplementary Material 8

Supplementary Material 9

Supplementary Material 10

Supplementary Material 11

Supplementary Material 12

## Data Availability

Data associated with this study are available at the NCBI Sequence Read Archive under BioProject accession PRJNA1226922.
